# Assessment of Interactions between Cisplatin and Two Histone Deacetylase Inhibitors in MCF7, T47D and MDA-MB-231 Human Breast Cancer Cell Lines – An Isobolographic Analysis

**DOI:** 10.1371/journal.pone.0143013

**Published:** 2015-11-18

**Authors:** Anna Wawruszak, Jarogniew J. Luszczki, Aneta Grabarska, Ewelina Gumbarewicz, Magdalena Dmoszynska-Graniczka, Krzysztof Polberg, Andrzej Stepulak

**Affiliations:** 1 Department of Biochemistry and Molecular Biology, Medical University of Lublin, Lublin, Poland; 2 Department of Pathophysiology, Medical University of Lublin, Lublin, Poland; 3 Isobolographic Analysis Laboratory, Institute of Rural Health, Lublin, Poland; 4 Department of Otolaryngology, MSW Hospital, Lublin, Poland; University of Quebec at Trois-Rivieres, CANADA

## Abstract

Histone deacetylase inhibitors (HDIs) are promising anticancer drugs, which inhibit proliferation of a wide variety of cancer cells including breast carcinoma cells. In the present study, we investigated the influence of valproic acid (VPA) and suberoylanilide hydroxamic acid (SAHA, vorinostat), alone or in combination with cisplatin (CDDP) on proliferation, induction of apoptosis and cell cycle progression in MCF7, T47D and MDA-MB-231 human breast carcinoma cell lines. The type of interaction between HDIs and CDDP was determined by an isobolographic analysis. The isobolographic analysis is a very precise and rigorous pharmacodynamic method, to determine the presence of synergism, addition or antagonism between different drugs with using variety of fixed dose ratios. Our experiments show that the combinations of CDDP with SAHA or VPA at a fixed-ratio of 1:1 exerted additive interaction in the viability of MCF7 cells, while in T47D cells there was a tendency to synergy. In contrast, sub-additive (antagonistic) interaction was observed for the combination of CDDP with VPA in MDA-MB-231 “triple-negative” (i.e. estrogen receptor negative, progesterone receptor negative, and HER-2 negative) human breast cancer cells, whereas combination of CDDP with SAHA in the same MDA-MB-231 cell line yielded additive interaction. Additionally, combined HDIs/CDDP treatment resulted in increase in apoptosis and cell cycle arrest in all tested breast cancer cell lines in comparison with a single therapy. In conclusion, the additive interaction of CDDP with SAHA or VPA suggests that HDIs could be combined with CDDP in order to optimize treatment regimen in some human breast cancers.

## Introduction

According to the American Cancer Society, breast cancer is the most frequent cancer (25%) among women diagnosed in 2012 [1]. The routine methods in the treatment of breast carcinoma are surgical resection, radiotherapy and chemotherapy. Many of cytostatic agents, such as anthracyclines, antimetabolites, alkylating agents and platinum-derivatives, including cisplatin (CDDP) have been tested in advanced breast cancer [2, 3]. Interest in platinum-based chemotherapy in breast cancer has been renewed, based on the hypothesis of greater susceptibility of triple-negative and BRCA1/2-mutant tumors to DNA-damaging chemotherapy agents [4]. Yet, standard chemotherapy with CDDP and other cytostatics is limited due to serious adverse-effects in treated patients and the occurrence of CDDP-resistance [5, 6]. Reducing CDDP-mediated cytotoxicity, or overcome CDDP-resistance with the concomitant use of other drugs, are of great importance. Recently, a new class of anticancer agents, histone deacetylase (HDAC) inhibitors (HDIs) has been introduced into the clinic. In 2006, suberoylanilide hydroxamic acid (SAHA, vorinostat, Zolizna^®^) has been registered by the U. S. Food and Drug Administration for treatment of cutaneous T-cell lymphoma (CTCL) [7]. Vorinostat has demonstrated activity in advanced multiple myeloma [8], advanced leukemia, myelodysplastic syndromes [9] and solid tumors, *inter alia* breast cancer, in clinical trials [10–12]. Valproic acid (VPA), for many years, has been an established drug in the treatment of epilepsy, manic-depressive disorders and migraines [13], more recently discovered also to have properties to inhibit the activity of HDACs [14]. Inhibition of HDACs causes increased level of acetylated histones, altering chromatin condensation and transcription, which in turn regulates expression of genes involved in cell cycle progression, cell differentiation, apoptotic pathways, autophagy, and mitotic cell death [15]. HDIs have shown anticancer activity against several types of tumor cells, both *in vitro* [16] and *in vivo* [17], with relatively low toxicity to normal cells [12]. Several molecular mechanisms have been proposed, which could be responsible for anti-cancer action of VPA, often depending on target cancer cell types. It has been reported that VPA induced cell cycle arrest by decreasing *CCND1* or *URG/URGCP* and increasing *p65* gene expression in SHSY5Y neuroblastoma cancer cells [18]. VPA caused decrease of cyclin D1 and increase in p21 and p27 expressions in LNCaP prostate cancer xenografts [19]. VPA-mediated upregulation of p21 was also observed in breast cancer cells [20] and in human cervical cancer xenograft model [21]. This action resulted in cellular senescence or terminal differentiation of head and neck squamous carcinoma cells [22]. Thereby, reintroduction of p21 expression, together with inhibition of cyclin D1 could be regarded as a more universal mechanism of VPA action on cancer cells. Several studies demonstrated that VPA can decrease activity/expression of proteins necessary for cancer progression, including anti-apoptotic protein survivin in neuroblastoma cells [23] or Bcl-2 on the mRNA and protein levels of in C6 glioma cells [24]. VPA could down-regulate of SMAD4, which resulted in reduced prostate cancer cell invasiveness probably trough the inhibition of the epithelial-mesenchymal transition [25]. VPA could also interfere with signaling pathways such as Notch in hepatocellular carcinoma [26], and ERK1/2 or Akt kinases in thyroid metastatic carcinoma [27]. Regarding breast cancer, VPA was shown to upregulate the metastasis suppressor Nm23H1 gene expression [28] or down-regulate surviving, which affected invasion and migration MDA-MB-231 breast cancer cells [29]. VPA treatment of MCF-7 breast cancer cells was associated with reduction of telomerase activity, increase in Bax/bcl-2 ratio [30], and decrease the expression of the invasiveness marker pS2 in ERα-positive breast cancer cells [31].

Similar to VPA, SAHA treatment of cancer cells resulted in inhibition of the cell cycle progression by decreasing expression of checkpoint regulatory proteins including cyclin-dependent kinases (CDKs) and cyclins, whereas increasing expression of cell cycle suppressors like p21 in ovarian carcinoma cells [32], esophageal squamous cancer cells [33], and breast MCF-7 cancer cells [34]. SAHA also triggered autophagic cell death through the inhibition of Akt/mTOR pathway signaling in glioblastoma stem cells [35], or in tamoxifen-resistant MCF-7 breast cancer cells by an unknown mechanism [36]. SAHA induced apoptosis by down-regulating survivin and Bcl-xL proteins in colon cancer cells [37] or by upregulation of molecules which belong to the TNF family, including soluble CD 137 receptor in the MDA-MB-231 breast cancer cell line. Recently, it has been demonstrated that SAHA epigenetically upregulates the expression of MHC class I-related chain molecules A and B (MICA and MICB) through regulating the expression of miR-17-92 cluster and MCM7 in hepatoma, resulting in an enhanced susceptibility of tumour cells to natural killer cell-mediated lysis [38]. The toxicity of standard anticancer agents can be overcome by using new chemotherapeutic combinations. The increase of the efficacy of therapy could be achieved through combined treatment with standard chemotherapeutics used in lower doses and non-toxic drugs that have different mechanism of action [39].

The type of interaction between HDIs and CDDP was determined by an isobolographic analysis. The isobolography is a very precise and rigorous pharmacodynamic method to determine the type of interactions between active agents, which exhibit a broad range of concentrations. The isobolographic analysis allows to precisely classify the observed interactions of drugs used in mixture at the fixed drug dose ratio (usually, 1:1). Theoretically, four types of interaction can be distinguished, as follows: supra-additivity (synergy), additivity, sub-additivity (relative antagonism) and infra-additivity (absolute antagonism) [40]. Our present study aimed to investigate the anti-cancer activity of CDDP in combination with SAHA or VPA in order to establish if this kind of treatment would enhance their anti-proliferative and proapototic activity in MCF7, T47D and MDA-MB-231 breast cancer cell lines.

## Materials and Methods

### Drugs

CDDP and VPA were purchased from Sigma (St. Louis, MO, USA), and dissolved in phosphate buffered saline (PBS) with Ca^2+^ and Mg^2+^ at 1 mg/ml and 100 mM concentrations as stock solutions, respectively. SAHA was purchased from Cayman Chemical (San Diego, CA, USA), and was dissolved in dimethyl sulfoxide (DMSO) at 10 mM concentration as stock solution. The drugs were diluted to the respective concentration with culture medium just before use.

### Cell lines

T47D (ATCC^®^ HTB-133^™^), MCF7 (ATCC^®^ HTB-22^™^) and MDA-MB-231 (ATCC^®^ HTB-26^™^) breast cancer cell lines were obtained from the American Type Culture Collection (Manassas, VA). All cancer cell lines were grown in DMEM/F12 culture medium (Sigma, St. Louis, MO, USA) supplemented with 10% FBS (Sigma), penicillin (Sigma) (100 IU/mL), and streptomycin (Sigma) (100 μg/mL) at 37°C in a humidified atmosphere with 5% CO_2_.

### Cell viability assay

MCF7, T47D and MDA-MB-231 cells were platted on 96-well microplates at a density of 3 × 10^4^ cells/ml. The cells were incubated in the presence of CDDP (0.01–10μg/ml), VPA (10–1000μg/ml), and SAHA (0.02–3μg/ml) for 96 h. After that, the cells were incubated for 3 h with the MTT (3-(4,5-dimethylthiazol-2-yl)-2,5-diphenyltetrazolium bromide) solution (5 mg/ml, Sigma). During the time MTT was metabolized by living cells to purple formazan crystals, which were later solubilized in SDS buffer (10% SDS in 0.01 N HCl) overnight. The optical density of the product was measured at 570 nm with the use of an Infinite M200 Pro microplate reader (Tecan, Männedorf, Switzerland).

The results of combined treatment CDDP and HDIs were analyzed according to isobolographic protocol. The drug doses were determined based on the IC_50_ values calculated from the previous cytotoxicity test.

### Isobolographic analysis of interactions

The percent inhibition of cell viability per dose of CDDP, SAHA and VPA administered alone and the dose-response relationship curves (DRRCs) for each investigated drug in three cancer cell lines (MCF7, T47D and MDA-MB-231) measured *in vitro* by the MTT assay were fitted using log-probit linear regression analysis according to Litchfield and Wilcoxon [41]. Subsequently, from the respective linear equations the median inhibitory concentrations (IC_50_s) of CDDP, SAHA or VPA administered alone were calculated. To precisely and correctly analyze the experimental data with isobolography, the test for parallelism of DRRCs for CDDP and SAHA or VPA based on the log-probit analysis was used [42, 43]. The test for parallelism was performed according to Litchfield and Wilcoxon [41], as described in detail in our previous study [44]. In this test, CDDP had its DRRC non-parallel to that of SAHA and VPA in three cancer cell lines (MCF7, T47D and MDA-MB-231) measured by the MTT assay. Isobolographic interactions between CDDP and SAHA or VPA in three cancer cell lines (MCF7, T47D and MDA-MB-231) were analyzed according to the methodology described by Grabovsky and Tallarida [45], Tallarida [46, 47], and Luszczki [42]. Based upon the IC_50_ values denoted experimentally for the drugs administered alone, median additive inhibitory concentrations of the mixture of CDDP with SAHA or VPA—i.e., concentrations of the mixture, which theoretically should inhibit cell viability in 50% (IC_50 add_) were calculated from two equations of additivity presented by Tallarida [46, 47]. For the lower line of additivity the equation at a 50% inhibitory effect for the combination of CDDP with SAHA is as follows: *y* = IC_50_SAHA_—[IC_50_SAHA_ / (IC_50_CDDP_ / *x*)^q/p^]; where *y*–is the concentration of SAHA; *x*–is the concentration of CDDP; *p* and *q*–are curve-fitting parameters (Hill coefficients) for SAHA and CDDP, respectively. Similarly, for the upper line of additivity the equation at a 50% inhibitory effect for the combination of CDDP with SAHA is: *y* = IC_50_SAHA_ [(IC_50_CDDP_—*x*)/ IC_50_CDDP_]^q/p^. To calculate the curve-fitting parameters (*p* and *q*), probits of response for SAHA and CDDP administered alone were transformed to % effect. Identical calculations were performed for the combination of CDDP with VPA. It is important to note that when two drugs produce maximal effect but are “heterodynamic” (i.e., have non-parallel DRRCs), the additivity is represented as an area bounded by two defined curves (lower and upper isoboles of additivity, for more detail see Tallarida) [46, 47]. The experimentally-derived IC_50_ values are statistically different if their points are placed outside this region. For supra-additivity (synergy), the experimentally-derived IC_50 mix_ points are placed below the area bounded by the lower and upper isoboles of additivity, and for sub-additivity (antagonism)–above this region [46, 47]. Proportions of CDDP and SAHA or VPA in the mixture were calculated only for the fixed-ratio combination of 1:1, and the mixtures of CDDP with SAHA or VPA concentrations were tested in three cancer cell lines (MCF7, T47D and MDA-MB-231) measured *in vitro* by the MTT assay. The evaluation of the experimentally-derived IC_50 mix_ at the fixed-ratio of 1:1 was based upon the concentration of the mixture inhibiting 50% of cell viability in three cancer cell lines (MCF7, T47D and MDA-MB-231) measured *in vitro* by the MTT assay. Finally, to determine the separate concentrations of CDDP and SAHA or VPA in the mixture, the IC_50 mix_ values were multiplied by the respective proportions of drugs (denoted for additive mixture). Further details regarding these concepts have been published elsewhere [42, 46, 47].

### Assessment of apoptosis

Cancer cells were placed on 6-well plates (Nunc) at a density of 1 x 10^5^/ml and then treated with VPA, SAHA and CDDP alone or in combination (SAHA/CDDP and VPA/CDDP) for 48 hours. After that, cells were harvested and washed twice with PBS. Next, cells were fixed and permeabilized using the Cytofix/ Cytoperm Solution according to the manufacturer’s instructions of PE Active Caspase-3 Apoptosis Kit (BD Pharmingen). Finally, cells were washed twice in the Perm/Wash Buffer prior to intracellular staining with PE-conjugated anti-active caspase-3 monoclonal rabbit antibodies. Labelled cells were analyzed by flow cytometer FACSCalibur (Becton Dickinson, San Jose, CA, USA), operating with CellQuest software to quantitatively assess the caspase-3 activity.

### Cell cycle analysis

Experiments were performed using the FACSCaliburTM flow cytometer (BD Biosciences). Breast cancer cell lines were treated with different concentrations of VPA, SAHA and CDDP alone or in combination (SAHA/CDDP and VPA/CDDP) for 48 hours and then fixed in ice-cold 80% ethanol at -20°C for 24 hours. After fixation, the cells were stained with propidium iodide utilizing the PI/RNase Staining Buffer (BD Biosciences) according to the manufacturer’s instructions. Acquisition rate was at least 60 events per second in low acquisition mode and at least 10,000 events were measured. Cell cycle analysis was performed by using flow cytometry analyzing software—Cylchred Version 1.0.2 for Windows (source: University of Wales) and WinMDI 2.9 for Windows (source: facs.scripps.edu/software.html). The cells were acquired and gated by using dot plot FL-2 Width (X) versus FL-2 Area (Y)-gate to exclude aggregates and analyzed in histograms displaying fluorescence 2-area (yellow-orange fluorescence: 585 nm).

### Statistical analysis

The IC_50_ and IC_50 mix_ values for CDDP, SAHA and VPA administered alone or in combination at the fixed-ratio of 1:1 were calculated by computer-assisted log-probit analysis according to Litchfield and Wilcoxon [41]. The experimentally-derived IC_50 mix_ values for the mixture of CDDP with SAHA or VPA were statistically compared with their respective theoretical additive IC_50_ add values by the use of unpaired Student’s t-test, according to Tallarida [48].

Preincubation and assessment of apoptosis results were presented using GraphPad Prism (one-way ANOVA; Turkey post-hoc testing). P<0.05 was considered to indicate a statistically significant difference. Results were presented as mean ± standard deviation (±SD) of the mean.

## Results

### Sequence independent anti-proliferation effects of CDDP, VPA and SAHA on breast cancer cells

**Fig 1:**
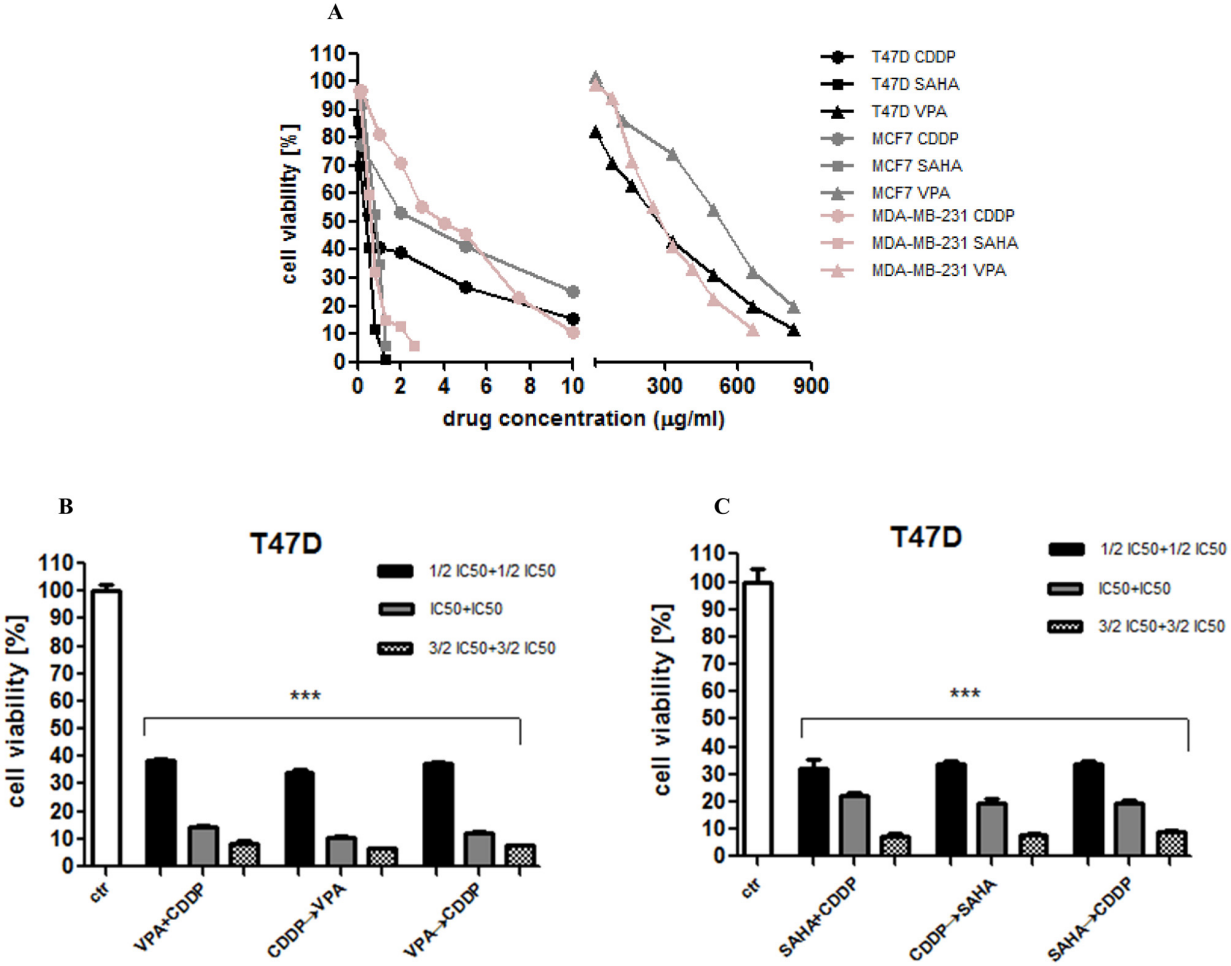
The anti-proliferative effects of HDIs and CDDP in breast cancer cell lines. (A) The anti-proliferative effects of VPA, SAHA, and CDDP on T47D, MCF7, MDA-MB-231 breast cancer cell lines after 96 h treatment with various concentration (0.1–1000 μg/ml) of active substances. The cell viability was measured by the MTT assay. (B) The anti-proliferative effects of VPA, SAHA, and CDDP on T47D breast cancer cell line. The cell viability was measured by the MTT assay. T47D cells were exposed to concomitant or sequential VPA and CDDP treatment using different ratios of the IC_50_ (1/2 IC_50_, IC_50_, 3/2 IC_50_). VPA+CDDP—valproic acid and cisplatin concomitant, CDDP→VPA—cisplatin (1h) followed by valproic acid, VPA→CDDP—valproic acid (1h) followed by cisplatin. Statistical analysis was performed using one-way ANOVA test (*** p<0.001). The results are means ± standard deviation (SD). (C) Examination of cell proliferation by MTT assay. T47D cells were exposed to concomitant or sequential SAHA and CDDP treatment using different ratios of the IC_50_ (1/2 IC_50_, IC_50_, 3/2 IC_50_). SAHA+CDDP—vorinostat and cisplatin concomitant, CDDP→SAHA cisplatin (1h) followed by vorinostat, SAHA→CDDP—vorinostat (1h) followed by cisplatin. The results are presented as mean ± standard deviation (SD). Statistical analysis was performed using one-way ANOVA test (*** p<0.001).

The anti-proliferative action of CDDP, VPA and SAHA was determined in the T47D, MCF7 and MDA-MB-231 breast cancer cell lines using the MTT assay in order to establish IC_50_ value for each analyzed compound in all cell lines. The IC_50_ values were the concentrations resulting in 50% cell growth inhibition by a 96 hours exposition to active agents as compared with control (untreated cells). In our study the dose-dependent growth inhibition effect of each compound was evident in all breast cancer cell lines (Fig 1A). We observed that T47D cells were the most sensitive to VPA, SAHA, or CDDP treatments among the all analyzed breast cancer cell lines (Fig 1A).

Next, we focused on the anti-proliferative activity of a combination of CDDP with HDIs. T47D cells were cultured in DMEM/F12 medium with combinations of different doses of CDDP/VPA and CDDP/SAHA in three different sequences: 1) treated with CDDP and VPA (or SAHA) at the same time for 96 h; 2) pretreated with CDDP 1h, followed by VPA (or SAHA) 96 h; 3) pretreated with VPA (or SAHA) 1h, followed by CDDP 96 h. As shown in Fig 1B and 1C the anti-proliferative activity of HDIs and CDDP in combination was sequence-independent.

### Anti-proliferative effects of SAHA administered singly and in combination with CDDP to the MCF7 cell line

**Fig 2:**
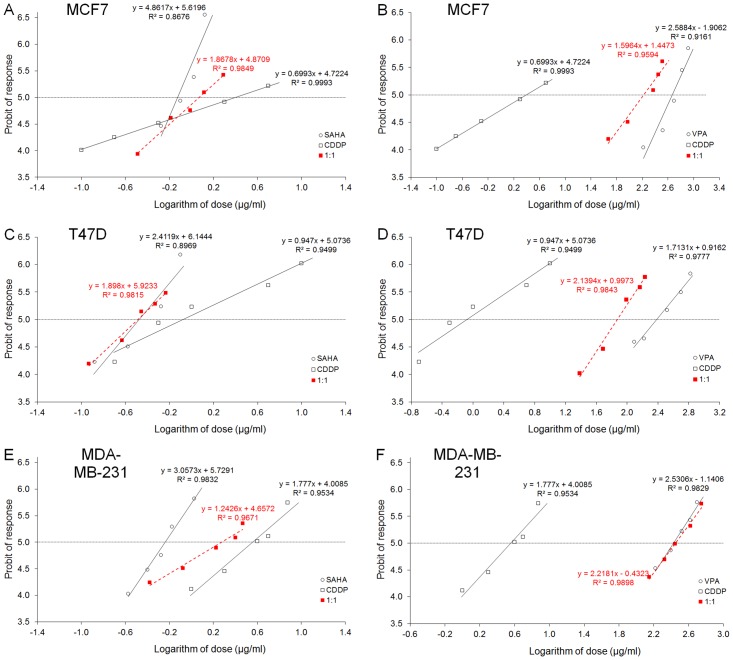
(A-F) Log-probit dose-response relationship curves (DRRCs) for HDIs and CDDP. DRRCs for cisplatin (CDDP), suberoylanilide hydroxamic acid (SAHA) and valproic acid (VPA) administered alone, and in combinations at the fixed-ratio of 1:1 (in red), illustrating the anti-proliferative effects of the drugs in three cancer cell lines (MCF7, T47D and MDA-MB-231) measured *in vitro* by the MTT assay. Doses of CDDP, SAHA and VPA administered separately and the mixture of the drugs at the fixed-ratio combination of 1:1 (in red) were transformed into logarithms, whereas the anti-proliferative effects produced by the drugs in three cancer cell lines (MCF7, T47D and MDA-MB-231) measured *in vitro* by the MTT assay were transformed into probits according to Litchfield and Wilcoxon (1949). Linear regression equations of DRRCs are presented on the graph; where y—is the probit of response, and x—is the logarithm (to the base 10) of a drug dose, R2 –coefficient of determination. Test for parallelism revealed that the experimentally determined DRRCs for CDDP, SAHA and VPA (administered alone) are not parallel to one another (for more details see Table 1.).

CDDP administered alone produced anti-proliferative effects in the MCF7 cells. The equation of DRRC for CDDP (y = 0.6993 x + 4.7224; Fig 2A), allowed the determination of the IC_50_ value for CDDP, which was 2.495 ± 1.223 μg/ml (Table 1). Similarly, SAHA administered alone produced anti-proliferative effects on MCF7 cells. The equation of DRRC for SAHA (y = 4.8617 x + 5.6196; Fig 2A) allowed the calculation of its IC_50_ value that amounted to 0.746 ± 0.068 μg/ml (Table 1). The test for parallelism of DRRCs between CDDP and SAHA revealed that the DRRCs of both compounds were non-parallel to each other (Table 1; Fig 2A). In this case, the combination of CDDP with SAHA at the fixed-ratio of 1:1 produced the anti-proliferative effects in the MCF7 cells and the IC_50 mix_ value calculated from the DRRC for the mixture of both compounds (y = 1.8678 x + 4.8709; Fig 2A) was 1.173 ± 0.295 μg/ml (Table 2).

**Table 1:** Anti-proliferative effects of CDDP, SAHA and VPA administered singly in three breast cancer cell lines (MCF7, T47D and MDA-MB-231) measured in vitro by the MTT assay.

Cell line	Drug	IC_50_ (μg/ml)	n	CFP	q/p	S.R.	F ratio S.R.	parallelism
MCF7	CDDP	2.495 ± 1.223	90	0.517 (q)	-	-	-	-
MCF7	SAHA	0.746 ± 0.068	54	1.924 (p1)	0.269	16.67	2.450	NP
MCF7	VPA	465.68 ± 68.96	72	1.210 (p2)	0.427	11.06	2.478	NP
T47D	CDDP	0.836 ± 0.359	64	0.869 (q)	-	-	-	-
T47D	SAHA	0.335 ± 0.065	48	1.988 (p1)	0.437	4.379	1.854	NP
T47D	VPA	242.0 ± 72.66	40	1.162 (p2)	0.748	2.967	2.170	NP
MDA-MB-231	CDDP	3.614 ± 0.740	80	1.031 (q)	-	-	-	-
MDA-MB-231	SAHA	0.577 ± 0.032	80	2.910 (p1)	0.354	1.721	1.372	NP
MDA-MB-231	VPA	267.0 ± 38.38	80	1.945 (p2)	0.530	1.471	1.466	NP

Results are presented as median inhibitory concentrations (IC_50_ values in μg/ml ± S.E.M.) of CDDP, SAHA and VPA administered singly with respect to their anti-proliferative effects on three cancer cell lines (MCF7, T47D and MDA-MB-231) measured in vitro by the MTT assay. n—total number of items used at concentrations whose expected anti-proliferative effects ranged between 4 and 6 probits (16% and 84%); CFP–(q and p) curve-fitting parameters; q/p—ratio of q and p values; S.R.–slope function ratio (S_CDDP_/S_SAHA_, S_CDDP_/S_VPA_); f ratio S.R.–factor for slope function ratio. Test for parallelism of two DRRCs was performed according to Litchfield and Wilcoxon (1949). It this case, if the slope function ratio (S.R.) value is higher than the factor for slope function ratio (f ratio S.R.) value, the examined two DRRCs are not parallel to each other (Litchfield and Wilcoxon 1949). NP—not parallel; All detailed calculations required to test the parallelism of two DRRCs were presented in the Appendix to the paper by Luszczki and Czuczwar (2006).

**Table 2:** Type I isobolographic analysis of interactions (for non-parallel DRRCs) between CDDP and SAHA or VPA at the fixed-ratio combination of 1:1 in three cancer cell lines (MCF7, T47D and MDA-MB-231) measured *in vitro* by the MTT assay.

Cell line	Combination	IC_50 mix_ (μg/ml)	n _mix_	^#^IC_50 add_ (μg/ml)	n _add_	^&^IC_50 add_ (μg/ml)	n _add_
MCF7	CDDP+SAHA	1.173 ± 0.295	48	0.926 ± 0.498	140	2.315 ± 0.691	140
MCF7	CDDP+VPA	168.1 ± 44.21	60	166.9 ± 63.06	158	301.2 ± 79.47	158
T47D	CDDP+SAHA	0.326 ± 0.072	60	0.422 ± 0.204	108	0.750 ± 0.221	108
T47D	CDP+VPA	74.30 ± 14.12	64	109.4 ± 39.01	100	133.5 ± 41.64	100
MDA-MB-231	CDDP+SAHA	1.888 ± 0.552	80	1.370 ± 0.546	156	2.819 ± 0.357	156
MDA-MB-231	CDDP+VPA	281.3 ± 40.11	80	106.0 ± 32.17 **	156	164.7 ± 36.91 *	156

Results are presented as median inhibitory concentrations (IC_50_ values in μg/ml ± S.E.M.) for two-drug mixtures, determined either experimentally (IC_50 mix_) or theoretically calculated (IC_50 add_) from the equations of additivity (Tallarida 2006, 2007), blocking proliferation in 50% of tested cells in three cancer cell lines (MCF7, T47D and MDA-MB-231) measured *in vitro* by the MTT assay. *n*_mix_—total number of items used at those concentrations whose expected anti-proliferative effects ranged between 16% and 84% (i.e., 4 and 6 probits) for the experimental mixture; *n*_add_—total number of items calculated for the additive mixture of the drugs examined (*n*_add_ = *n*__CDDP_ + *n*__SAHA_− 4) or (*n*_add_ = *n*__CDDP_ + *n*__VPA_− 4);

^#–^IC_50 add_ value calculated from the equation for the lower line of additivity;

^&–^IC_50 add_ value calculated from the equation for the upper line of additivity.

Statistical evaluation of data was performed with unpaired Student’s *t*-test according to Tallarida (2000). There was no statistical difference between the IC_50 mix_ and IC_50 add_ values with unpaired Student’s *t*-test and thus, the analyzed interactions were additive in two cancer cell lines (MCF7 and T47D) as measured by the MTT assay *in vitro*. Similarly, no significant difference was observed between the IC_50 mix_ and IC_50 add_ values for the combination of CDDP with SAHA in the MDA-MB-231 cell line. In contrast, the IC_50 mix_ value for the combination of CDDP with VPA was significantly higher than its corresponding IC_50 add_ values (P<0.05 and P<0.01), suggesting sub-additive (antagonistic) anti-proliferative effects of the combination in the MDA-MB-231 cell line.

*P<0.05 and **P<0.01 vs the respective IC_50 mix_ value.

### Anti-proliferative effects of VPA administered singly and in combination with CDDP in the MCF7 cell line

VPA administered alone reduced the proliferation of MCF7 cells. The equation of DRRC for VPA (y = 2.5884 x—1.9062; Fig 2B) allowed the calculation of its IC_50_ value that amounted to 465.68 ± 68.96 μg/ml (Table 1). The test for parallelism of DRRCs between CDDP and VPA revealed that the DRRCs of both compounds were non-parallel to each other (Table 1; Fig 2B). In this case, the combination of CDDP with VPA at the fixed-ratio of 1:1 produced the anti-proliferative effects in the MCF7 cells and the IC_50 mix_ value calculated from the DRRC for the mixture of both compounds (y = 1.5964 x + 1.4473; Fig 2B) was 168.1 ± 44.21 μg/ml (Table 2).

### Anti-proliferative effects of SAHA administered singly and in combination with CDDP on T47D cells

CDDP administered alone produced anti-proliferative effects on T47D cells. The equation of DRRC for CDDP (y = 0.947 x + 5.0736; Fig 2C), allowed the determination of the IC_50_ value for CDDP, which was 0.836 ± 0.359 μg/ml (Table 1). Similarly, SAHA administered alone produced anti-proliferative effects on T47D cells. The equation of DRRC for SAHA (y = 2.4119 x + 6.1444; Fig 2C) allowed the calculation of its IC_50_ value that amounted to 0.335 ± 0.065 μg/ml (Table 1). The test for parallelism of DRRCs between CDDP and SAHA revealed that the DRRCs of both compounds were non-parallel to each other (Table 1; Fig 2C). In this case, the combination of CDDP with SAHA at the fixed-ratio of 1:1 produced the anti-proliferative effects on T47D cells and the IC_50 mix_ value calculated from the DRRC for the mixture of both compounds (y = 1.898 x + 5.9233; Fig 2C) was 0.326 ± 0.072 μg/ml (Table 2).

### Anti-proliferative effects of VPA administered singly and in combination with CDDP on T47D cells

An anti-proliferative effect in the T47D cells by VPA administered alone was observed. The equation of DRRC for VPA (y = 1.7131 x + 0.9162; Fig 2D) allowed the calculation of its IC_50_ value that amounted to 242.0 ± 72.66 μg/ml (Table 1). The test for parallelism of DRRCs between CDDP and VPA revealed that the DRRCs of both compounds were non-parallel to each other (Table 1; Fig 2D). In this case, the combination of CDDP with VPA at the fixed-ratio of 1:1 produced the anti-proliferative effects in the T47D cell line and the IC_50 mix_ value calculated from the DRRC for the mixture of both compounds (y = 2.1394 x + 0.9973; Fig 2D) was 74.30 ± 14.12 μg/ml (Table 2).

### Isobolographic analysis of interactions between CDDP and SAHA in the MCF7 cells

**Fig 3:**
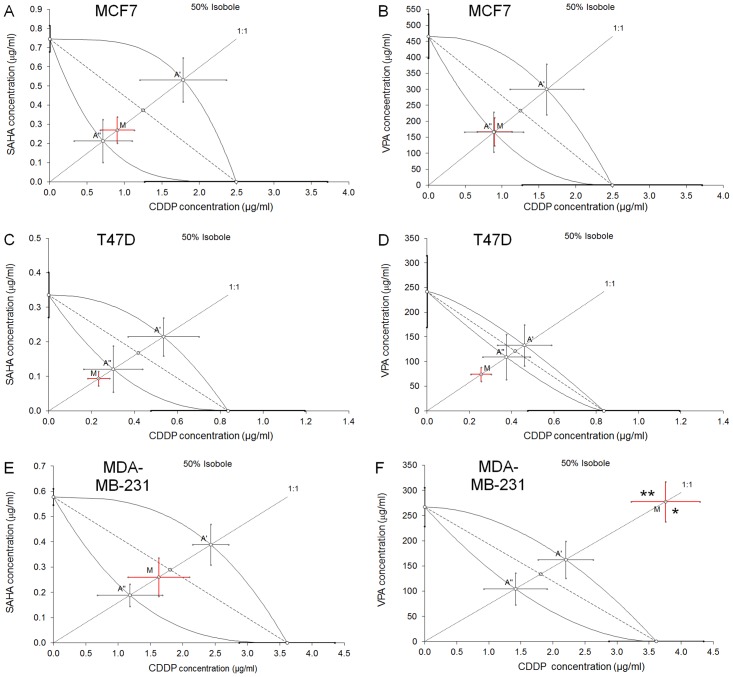
(A-F) Isobolographic analysis of interactions between HDIs and CDDP. Isobolograms showing additive interactions between cisplatin (CDDP), suberoylanilide hydroxamic acid (SAHA) and valproic acid (VPA) with respect to their anti-proliferative effects on three cancer cell lines (MCF7, T47D and MDA-MB-231) measured *in vitro* by the MTT assay. The median inhibitory concentrations (IC_50_) for CDDP, SAHA and VPA are plotted graphically on the X- and Y-axes, respectively. The solid lines on the X and Y axes represent the S.E.M. for the IC_50_ values for the studied drugs administered alone. The lower and upper isoboles of additivity represent the curves connecting the IC_50_ values for CDDP and SAHA or VPA administered alone. The dotted line starting from the point (0, 0) corresponds to the fixed-ratio of 1:1 for the combination of CDDP with SAHA or VPA. The diagonal dashed line connects the IC_50_ for CDDP and SAHA or VPA on the X- and Y-axes. The points A’ and A” depict the theoretically calculated IC_50 add_ values for both, lower and upper isoboles of additivity. The point M represents the experimentally-derived IC_50 mix_ value for total dose of the mixture expressed as proportions of CDDP and SAHA or VPA that produced a 50% anti-proliferative effect (50% isobole) in three cancer cell lines (MCF7, T47D and MDA-MB-231) measured *in vitro* by the MTT assay. On the graph, the S.E.M. values are presented as horizontal and vertical error bars for every IC_50_ value. The experimentally-derived IC_50 mix_ value is placed close to the point A”, indicating additive interaction between CDDP and SAHA or VPA in three cancer cell lines (MCF7, T47D and MDA-MB-231) measured in vitro by the MTT assay.

The isobolographic analysis of interaction for non-parallel DRRCs revealed that the mixture of CDDP with SAHA at the fixed-ratio of 1:1 exerted additive interaction in the MCF7 cells (Fig 3A). The experimentally derived IC_50 mix_ value for this fixed-ratio combination was 1.173 μg/ml, whereas the additively calculated IC_50 add_ values were 0.926 μg/ml (for the lower IC_50 add_) and 2.315 μg/ml (for the upper IC_50 add_; Table 2). Thus, the IC_50 mix_ value did not significantly differ from the IC_50 add_ values (Table 2, Fig 3A).

### Isobolographic analysis of interactions between CDDP and VPA on MCF7 cells

The isobolographic analysis of interaction for non-parallel DRRCs revealed that the mixture of CDDP with VPA at the fixed-ratio of 1:1 exerted additive interaction on MCF7 cells (Fig 3B). The experimentally derived IC_50 mix_ value for this fixed-ratio combination was 168.1 μg/ml, whereas the additively calculated IC_50 add_ values were 166.9 μg/ml (for the lower IC_50 add_) and 301.2 μg/ml (for the upper IC_50 add_; Table 2). Thus, the IC_50 mix_ value did not significantly differ from the IC_50 add_ values (Table 2, Fig 3B).

### Isobolographic analysis of interactions between CDDP and SAHA on T47D cells

The isobolographic analysis of interaction for non-parallel DRRCs revealed that the mixture of CDDP with SAHA at the fixed-ratio of 1:1 exerted additive interaction on T47D cells (Fig 3C). The experimentally derived IC_50 mix_ value for this fixed-ratio combination was 0.326 μg/ml, whereas the additively calculated IC_50 add_ values were 0.422 μg/ml (for the lower IC_50 add_) and 0.750 μg/ml (for the upper IC_50 add_; Table 2). Thus, the IC_50 mix_ value did not significantly differ from the IC_50 add_ values, although a tendency towards synergy was observed (Table 2, Fig 3C).

### Isobolographic analysis of interactions between CDDP and VPA on T47D cells

The isobolographic analysis of interaction for non-parallel DRRCs revealed that the mixture of CDDP with VPA at the fixed-ratio of 1:1 exerted additive interaction in the T47D cell line (Fig 3D). The experimentally derived IC_50 mix_ value for this fixed-ratio combination was 74.30 μg/ml, whereas the additively calculated IC_50 add_ values were 109.4 μg/ml (for the lower IC_50 add_) and 133.5 μg/ml (for the upper IC_50 add_; Table 2). Thus, the IC_50 mix_ value did not significantly differ from the IC_50 add_ values, although a tendency towards synergy was observed (Table 2, Fig 3D).

### Anti-proliferative effects of SAHA administered singly and in combination with CDDP on MDA-MB-231 cells

CDDP administered alone produced anti-proliferative effects on MDA-MB-231 cells. The equation of DRRC for CDDP (y = 1.777 x + 4.0085; Fig 3E), allowed the determination of the IC_50_ value for CDDP, which was 3.614 ± 0.740 μg/ml (Table 1). Similarly, SAHA administered alone produced anti-proliferative effects on MDA-MB-231 cells. The equation of DRRC for SAHA (y = 3.0573 x + 5.7291; Fig 3E) allowed the calculation of its IC_50_ value that amounted to 0.577 ± 0.032 μg/ml (Table 1). The test for parallelism of DRRCs between CDDP and SAHA revealed that the DRRCs of both compounds were non-parallel to each other (Table 1; Fig 3E). In this case, the combination of CDDP with SAHA at the fixed-ratio of 1:1 produced the anti-proliferative effects on MDA-MB-231 cells and the IC_50 mix_ value calculated from the DRRC for the mixture of both compounds (y = 1.2426 x + 4.6572; Fig 3E) was 1.888 ± 0.552 μg/ml (Table 2).

### Anti-proliferative effects of VPA administered singly and in combination with CDDP on MDA-MB-231 cells

An anti-proliferative effect in the MDA-MB-231 cells by VPA administered alone was observed. The equation of DRRC for VPA (y = 2.5306 x—1.1406; Fig 3F) allowed the calculation of its IC_50_ value that amounted to 267.0 ± 38.38 μg/ml (Table 1). The test for parallelism of DRRCs between CDDP and VPA revealed that the DRRCs of both compounds were non-parallel to each other (Table 1; Fig 3F). In this case, the combination of CDDP with VPA at the fixed-ratio of 1:1 produced the anti-proliferative effects in the MDA-MB-231 cell line and the IC_50 mix_ value calculated from the DRRC for the mixture of both compounds (y = 2.2181 x—0.4323; Fig 3F) was 281.3 ± 40.11 μg/ml (Table 2).

### Isobolographic analysis of interactions between CDDP and SAHA on MDA-MB-231 cells

The isobolographic analysis of interaction for non-parallel DRRCs revealed that the mixture of CDDP with SAHA at the fixed-ratio of 1:1 exerted additive interaction on MDA-MB-231 cells (Fig 3E). The experimentally derived IC_50 mix_ value for this fixed-ratio combination was 1.888 μg/ml, whereas the additively calculated IC_50 add_ values were 1.370 μg/ml (for the lower IC_50 add_) and 2.819 μg/ml (for the upper IC_50 add_; Table 2). Thus, the IC_50 mix_ value did not significantly differ from the IC_50 add_ values (Table 2, Fig 3E).

### Isobolographic analysis of interactions between CDDP and VPA on MDA-MB-231 cells

The isobolographic analysis of interaction for non-parallel DRRCs revealed that the mixture of CDDP with VPA at the fixed-ratio of 1:1 exerted sub-additive (antagonistic) interaction in the MDA-MB-231 cell line (Fig 3F). In this case, the experimentally derived IC_50 mix_ value for this fixed-ratio combination was 281.3 μg/ml, whereas the additively calculated IC_50 add_ values were 106.0 μg/ml (for the lower IC_50 add_) and 164.7 μg/ml (for the upper IC_50 add_; Table 2). Thus, the IC_50 mix_ value significantly differed from the IC_50 add_ values (P<0.05 and P<0.01) indicating sub-additive (antagonistic) interaction between the tested compounds (Table 2, Fig 3F).

### Enhancement of CDDP induced apoptosis by HDAC inhibitors

**Fig 4:**
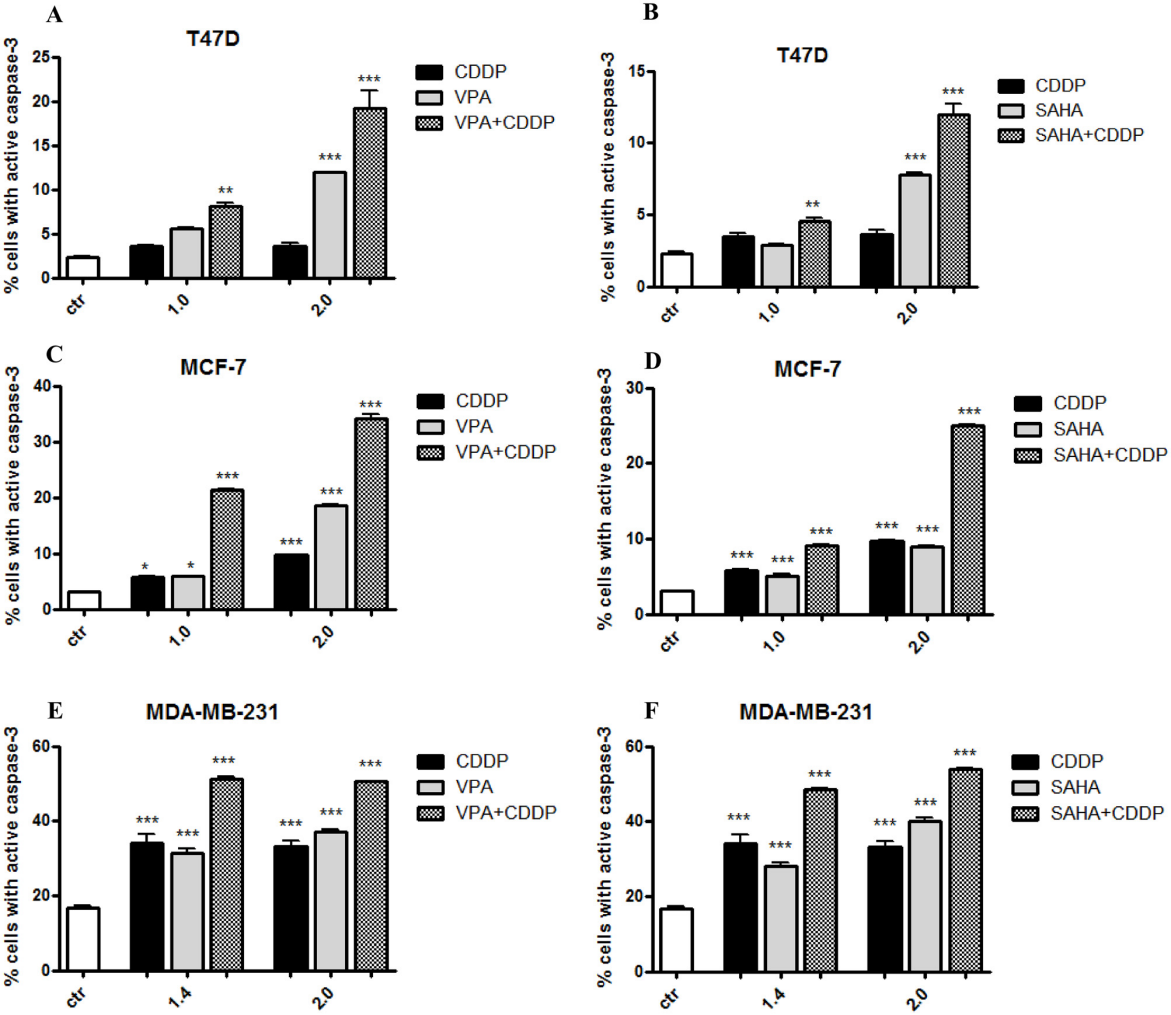
(A-F) Effects of HDIs and CDDP on caspase-3 activation in breast cancer cells. Apoptotic changes induced by VPA or SAHA and CDDP alone or in combinations. Three breast cancer cell lines (T47D, MCF7, MDA-MB-231) were cultivated for 48 h with different doses of active drugs and their mixtures (2.0 = 100% IC_50,_ 1.4 = 70% IC_50,_ 1.0 = 50% IC_50,_) and analyzed by flow cytometry. The values present the percentage of the cells with active caspase-3. The results are depicted as means ± SD, n = 5 from 5 separate experiments. Statistical analysis was performed using one-way ANOVA test (*** p<0.001, ** p<0,01, * p<0,05).

Obtained results demonstrated that VPA and SAHA alone or in combination with CDDP after 48 h incubation caused a dose-dependent increase in number of apoptotic cells in all analyzed breast cancer cell lines. HDIs increased cisplatin induced apoptosis in these three cell lines. Treatment with SAHA or VPA alone in T47D, MCF7 and MDA-MB-231 cells increased the numbers of apoptotic cells several-fold above the control level. When T47D and MDA-MB-231 cells were incubated with CDDP alone, cellular apoptosis was induced, but not in a dose-dependent manner. However, when combination of HDIs and CDDP were applied dose-dependent increase in the degree of apoptosis was evident. As shown in Fig 4C treatment of MCF7 cells with 50% IC_50_ (1.0 in Fig 4C) VPA and CDDP alone induced apoptosis in 5.81% and 5.89% cells, respectively. The percentage of apoptotic cells increased to 21.4% after simultaneous treatment with CDDP and VPA. Doubling the doses of VPA and CDDP (IC_50_ = 2.0) caused increase of apoptosis to 34.17% (Fig 4C). Similar data were obtained for combined treatment of VPA and CDDP, or SAHA and CDDP in MCF7 and T47D cell lines. (Fig 4A–4D). The induction of apoptosis after combined treatment was even more evident in MDA-MB-231 triple negative breast cancer cell line. Thereby, this analysis revealed that HDIs in combination with CDDP produced a significant apoptotic effect (Fig 4A–4F).

### Effects of CDDP/HDIs on cell cycle progression

**Fig 5:**
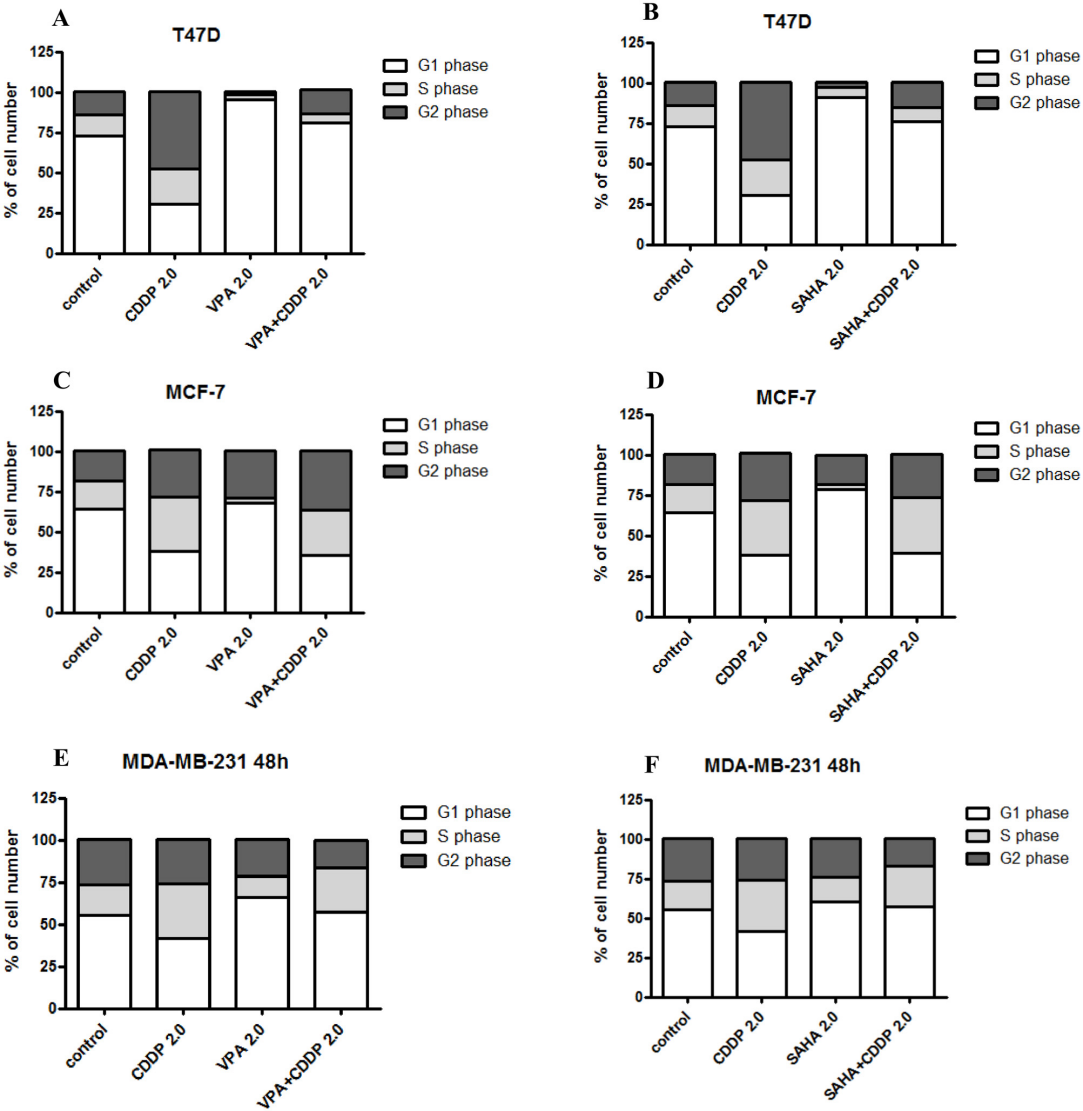
(A-F) Effects of CDDP and HDIs on cell cycle progression in breast cancer cells. Effect of HDIs alone and in combination with CDDP on cell cycle in T47D, MCF7 and MDA-MB-231 cells. The cell lines were cultivated for 48 h with VPA or SAHA and CDDP (2.0 = 100% IC_50_) and analyzed by flow cytometry. The results are presented as mean + SD, n = 10 from five separate experiments.

Effects of VPA, SAHA and CDDP treatment alone or in combination on the cell cycle progression was examined in three breast cancer cell lines (T47D, MCF7, MDA-MB-231). FACS analysis of PI-stained cells indicated that incubation breast cancer cells with SAHA or VPA at relatively high concentrations (2.0 = IC_50_) for 48h led to accumulation of cells in the G1 phase of the cell cycle corresponding with the cell reduction in the G2 phase. This effect was more pronounced in T47D cell line comparing to MCF-7 and MDA-MB-231 cells. Incubation with CDDP resulted in increase of cells number in G2 phase, followed by the reduction of cell number in G1 phase in T47D and to a lesser extent in MCF-7 cells. The increase of cell number in G2 phase in MDA-MB-231 cell line was not apparent. Simultaneous exposure to HDIs and CDDP maintained HDIs induced G1 arrest in T47D cells (Fig 5A and 5B). Incubation of MCF7 cells with HDIs and CDDP resulted in cell cycle inhibition pattern similar to that induced by CDDP alone (Fig 5C and 5D).

## Discussion

In spite of emerging new therapeutic options, such as monoclonal antibodies or small molecule tyrosine kinase inhibitors, chemotherapy on different types of cancers is not as effective as expected. Therefore, on the one hand, more effective chemotherapeutic agents, such as HDIs are being looked for; while on the other hand, combinations of established anti-cancer drugs and new cytostatics are being tested [49, 50]. Recently, a renewed interest in platinum-based chemotherapy in breast cancer is observed, especially in the treatment of triple-negative (TNBC) and BRCA1/2-mutant tumors [4]. They are characterized by a very aggressive growth associated with a high risk of relapses, metastasis, and worse response to therapy than hormone positive-receptor types of breast cancer [51]. Thus, we tested if HDI/CDDP drugs combinations would be more effective for treatment of different types of breast cancer cells *in vitro* using isobolographic method. Synergistic action of HDIs and other chemotherapeutic agents like CDDP have allowed to reduce the doses of cytostatics in order to achieve similar or better therapeutic effects for head and neck squamous cell carcinomas [49, 50]. A synergistic action by SAHA and CDDP in oral squamous cell carcinoma, and breast cancer cells *in vitro*, as measured by MTT and TUNEL assays, has previously been reported [52, 53].

The isobolographic analysis is the best method used to characterize the type of interactions between drugs in both *in vivo* and *in vitro* studies [40, 54]. This very efficient method is rarely used to establish drug-drug interaction in cancer-related studies. Instead, usually simple-type associations between tested compounds are presented, where only one or few random chosen doses are selected, without precise method of drug-type interaction analysis [53, 55–57].

In our studies, on the basis on the isobolographic analysis, we showed the additivity, with a tendency towards synergy, between the CDDP and HDIs (VPA and SAHA) in inhibiting cancer cell proliferation in T47D and MCF-7 breast cancer cells by means of MTT assay. Only combination of CDDP with VPA at the ratio of 1:1 induced sub-additive (antagonistic) interactions in MDA-MB-231 cells, while CDDP and SAHA induced synergy-like interactions in the same cell line. The mechanism by which this phenomenon of differential response occurs is not known, but may involve alterations in the HDACs activities mediated by HDIs. SAHA and VPA show strong inhibitory potency against HDAC1, 2, 3, but VPA weakly inhibits activity of HDAC6, compared with SAHA [58]. Taking into consideration that HDAC6 plays an important role in progression of human breast cancer [59], this could explain possible differences between anticancer activity of SAHA/CDDP and VPA/CDDP combinations in triple negative breast cancer cells. Several nuclear proteins are substrates of HDAC6, including the transcription factor forkhead boxp3 (FOXP3) and the DNA repair factor KU70. HDAC6 is also recruited by RNA polymerase-II to chromatin to reset acetylation/deacetylation cycles, enabling transcription and carries cytotoxic poly-ubiquitinylated proteins into autophagosomes [60]. These plethora of different mechanisms of HDAC6 actions and its potential inhibition by SAHA may be responsible for observed differences. The tendency towards additivity or synergy, as reported for the combination of CDDP with SAHA or VPA, on luminal (MCF7 and T47D) human breast cancer cells, may result in possible overlapping mechanisms of action of both types of compounds.

According to the cell cycle progression analysis we observed, that cell cycle changes induced by single or combined HDIs/CDDP treatment depended on the cell line used. In contrast, combined CDDP/HDIs treatment markedly enhanced apoptosis of breast cancer cells, including triple negative MDA-MB-231 cells. HDIs are able to induce apoptosis or inhibit cell cycle by regulating expression of several genes [61, 62]. VPA induces apoptosis through up-regulation of Bak and down-regulation of Bcl-2 genes expression in MCF7 breast cancer cells [63]. Almost all hitherto known, HDIs are able to activate the transcription of the CDKN1A gene encoding the p21WAF1 [64], an inhibitor of CDK2, the primary enzyme involved in the regulation of the cell cycle, and inhibit the expression of cyclin D1, A, E. Additionally, SAHA has been shown to down-regulate genes modulating the G1/S (cyclin D) and G2/M transition (cyclin B1) [65], which results in the inhibition of the cell cycle progression. Platinum complexes (including CDDP and derivatives) inhibit DNA replication by creating bonds intra-strand (binding to N atoms (7) neighboring guanines) [66]. Similarly to HDI, CDDP induces the process of apoptosis, through decreased of Bcl-2 gene expression [67]. It is also related to inhibition of DNA synthesis followed by cells arrest in G1, S, G2/M phases of the cell cycle [68, 69].

In our studies, concomitant administration of CDDP and HDIs allowed to reduce the doses of CDDP while achieving a similar anti-proliferative effect. HDIs, unlike other cytostatic-type compounds, have been reported to have much lower toxicity on normal cells, in contrast to cancer cells [70], and to develop minor adverse-effects in patients as demonstrated in clinical trials phases I and II [68]. The exact mechanism responsible for these phenomena is not fully elucidated. It seems that in contrast to normal cells, cancer cells cannot overcome the changes induced by HDIs, as shown using normal (HFS) or lung cancer cell lines (A549, LNCaP) [70]. It has been also demonstrated that VPA resensitized cisplatin-resistant ovarian [71] and neuroblastoma cancer cells [72] to CDDP treatment, thereby enabling to overcome the serious clinical problem of drug-resistance in the cancer chemotherapy.

HDI may be clinically important since doses of SAHA inducing growth arrest of the breast cancer cell line at 50% (IC_50_ = 1.27μM for T47D, IC_50_ = 2.6μM for MDA-MB-231 and IC_50_ = 3.3μM for MCF7 cells) induction of apoptosis were within the permitted and safe doses (0,43–5μM) used in clinics [73]. Additionally, VPA strongly repressed the survival of the breast cancer cells, however the VPA dose displaying antiproliferative effects exceeded the maximal dose used on patients (0,7 mM) [74]. Consequenlty, our results show that VPA can be used to design a combined therapy based on cisplatin. Taking under the consideration that CDDP induses strong side effects, lowering the dose of this cytostatic combined with the additional application of VPA at already clinically used doses appears to be a promising alternative therapeutic strategy.

These results seem to be promising and may create a positive attitude to research this type of interaction *in vivo*, in an animal model. Anticancer properties of SAHA and VPA make these drugs a subject of clinical interest as a new approach to treat breast cancers.
